# The socio-economic transition and health professions education in Mongolia: a qualitative study

**DOI:** 10.1186/s12962-021-00269-5

**Published:** 2021-03-07

**Authors:** Nomin Amgalan, Jwa-Seop Shin, Seung-Hee Lee, Oyungoo Badamdorj, Oyungerel Ravjir, Hyun Bae Yoon

**Affiliations:** 1 Department of Medical Education, Seoul National University College of Medicine, 103 Daehak-ro, Jongno-gu, Seoul, 03080 Korea; 2 Division of Educational Policy and Management, Mongolian National University of Medical Sciences, Ulaanbaatar, Mongolia; 3 Department of Infectious Diseases, Mongolian National University of Medical Sciences, Ulaanbaatar, Mongolia; 4 Office of Medical Education, Seoul National University College of Medicine, Seoul, Korea

**Keywords:** Transition economy, Health Professions Education, Mongolia

## Abstract

**Background:**

Former socialist countries have undergone a socio-economic transition in recent decades. New challenges for the healthcare system have arisen in the transition economy, leading to demands for better management and development of the health professions. However, few studies have explored the effects of this transition on health professions education. Thus, we investigated the effects of the socio-economic transition on the health professions education system in Mongolia, a transition economy country, and to identify changes in requirements.

**Methods:**

We used a multi-level perspective to explore the effects of the transition, including the input, process, and output levels of the health professions education system. The input level refers to planning and management, the process level refers to the actual delivery of educational services, and the output level refers to issues related to the health professionals, produced by the system. This study utilized a qualitative research design, including document review and interviews with local representatives. Content analysis and the constant comparative method were used for data analysis.

**Results:**

We explored tensions in the three levels of the health professions education system. First, medical schools attained academic authority for planning and management without proper regulation and financial support. The government sets tuition fees, which are the only financial resource of medical schools; thus, medical schools attempt to enroll more students in order to adapt to the market environment. Second, the quality of educational services varies across institutions due to the absence of a core curriculum and differences in the learning environment. After the transition, the number of private medical schools rapidly increased without quality control, while hospitals started their own specialized training programs. Third, health professionals are struggling to maintain their professional values and development in the market environment. Fixed salaries lead to a lack of motivation, and quality evaluation measures more likely reflect government control than quality improvement.

**Conclusions:**

Mongolia continues to face the consequences of the socio-economic transition. Medical schools’ lack of financial authority, the varying quality of educational services, and poor professional development are the major adverse effects. Finding external financial support, developing a core curriculum, and reforming a payment system are recommended.

## Background

The socio-economic transition refers to the shift from a centrally planned economy to a market economy. In the first half of the 20th century, the majority of countries with a centrally planned economy adopted socialist systems, a great achievement of which was the provision of free social services [1]. Nevertheless, these systems eventually became economically inefficient, and many socialist countries started transitioning to the market system in the mid-1980s [2]. Former socialist countries faced deep macro-economic crises, such as hyperinflation, rising unemployment, lack of a functioning legal system, and corruption [3]. Most former socialist countries followed a similar strategic pattern of macro-economic reforms, including decentralization and privatization. The transition processes and recovery levels varied among countries as a result of when the transition began and the specific changes that were made [4].

The health sector became extremely under-funded; past achievements were threatened. Health facilities became non-operational due to the depleted government budget, and the quality of services deteriorated [5]. Many researchers have investigated the effects of the transition and reforms, including in the health sector. For instance, a study in China showed that healthcare institutions had difficulties recruiting health professionals in the public sector [6]. The same problems arose in Ghana, where the limited local authority for human resource management has adversely affected the development of health professionals [7]. A study in Cambodia showed that the health financing reforms had varying level of effectiveness across the provinces; some factors for efficiency were more dependent on the policy-makers [8]. Some researchers have recommended capacity changes of the health professions as a way to implement effective health reforms in Myanmar [9], while other researchers suggested focusing on a supply-side system of health reforms in China, Mexico, and Vietnam [10].

Mongolia had a socialist system for almost seven decades, and embarked upon the political and economic transition in the early 1990s [11]. Mongolia followed the same strategies as the Czech Republic, Poland, and Romania, and its economic situation smoothly developed and quickly recovered compared to other countries in the Baltic regions, the Commonwealth of Independent States, and Central and Eastern Asia [3]. Currently, the health expenditure accounted as 7.2% of government total expenditure in Mongolia, and the health care is provided by the state and private sectors (Table 1) [12, 13]. Many international and local researchers have conducted studies and evaluations of the socio-economic transition in Mongolia, highlighting the challenges facing the health professions education (HPE) system. For instance, competency mismatches, poor teamwork, and lack of capacity of health professionals are major issues in the healthcare system [14].

**Table 1:** General information of the Mongolian health system

Indicators	2000	2010	2019
Health expenditure			
Current health expenditure, million tugrugs	45 951.6	250 264.7	823 888.9
Percentage of health expenditure in general government total expenditure	10.7	8.1	7.2
Health expenditure per capita, thousand tugrugs	19.2	91.4	258.3
Health personnel			
Total health employees	31,507	39,608	54,687
Physicians	6498	7497	11,788
Mid-level medical personnels (nurse, midwive, technician, etc.)	13,481	15,858	20,799
Health facilities			
Total health institutions	1580	2388	3824
Number of state health institions	396	391	393
Number of private health institutions	1184	1997	3431
Total hospital beds	17,974	17,821	25,661
Hospital beds, 1000 population	7.5	7	8

Source: Mongolian Statistical Yearbook-2003, 2019, National Statistics Office of Mongolia

Transition economy countries are struggling to maintain a satisfactory quality of healthcare services due to the challenges facing the HPE system. The experiences of other countries have shown that the socio-economic transition leads to new demands for the better management and development of the health professions. Mongolia is an appropriate example of a transition economy country for exploring the effects of the transition. Because Mongolia had one of the longest socialist histories in the world, it continues to experience the transition economy. To obtain some insights from the case of Mongolia, we investigated the effects of the socio-economic transition on the HPE system and sought to identify changing requirements in transition economy countries.

## Methods

### Multi-level perspective

We used the multi-level perspective (MLP) approach, which asserts that developments at various levels in social organizations are linked with each other. Thus, the nature of these linkages should be investigated to understand what occurs at specific levels. In this framework, the concept of linkage denotes the transmission of communication or materials from one level to another [15]. We utilized the three level input-process-output model, which is widely used in different fields, but has a consistent logic based on its purpose of measuring the quality of a system. The input level focuses on a variety of aspects such as plans, policies, financing, and participants’ characteristics. The process level refers to the actual delivery of educational services. The output level denotes the desired result of the educational system, which in this case relates to issues facing the health professions [16]. Moreover, time linkages have emerged as a focus in resent studies on historical development. The study of time linkages could provide insights on whether certain changes support sustainable development in a social organization [15]. The data collection methods used in this study were document review and interviews.

### Document review

We searched for documents published in English and Mongolian through international websites such as PubMed, Google Scholar, and World Health Organization and the local websites of the Ministry of Health, Mongolian Medical Education Association, and medical universities. We used the terms “transition economy,” “socialist system,” “market system,” “reforms,” “healthcare system,” “human resources for health,” and “health professions education” in combination with the term “Mongolia.” Some documents in local sources were published only in printed form; thus one of the researchers visited the central library in Mongolia to collect them.

Two researchers independently screened the titles, abstracts, and summaries of documents in English, while two local researchers screened the documents in Mongolian in the same way. From a total of 86 documents, we selected those that contained information on the three levels in the MLP framework. Documents with limited information on healthcare and HPE systems in the context of the transition and reforms were excluded. We retained 14 documents for the final analysis, and provided PRISMA statement for detailed information (Appendix 1). We categorized them as English and Mongolian documents; nine were published online and five were print-only documents (Table 2).

**Table 2:** Document review

	Published year	Title	Author	Document type
English documents	1994	Transition from socialism and the financing of higher education: the case of Mongolia	Mark B et al	Article
	1995	Poverty and the transition to a market economy in Mongolia	Keith G	Book
	2001	Mongolia’s system-wide health reforms: lessons for other developing countries	Michael OM, Don H	Article
	2007	Education in Mongolia: The difficulties and achievements of the period of transition	Suprunova L L	Article
	2013	Health systems transition in Mongolia: Transition	World Health Organization	Report
	2017	Role of emerging private hospitals in a post-Soviet mixed health system: a mixed methods comparative study of private and public hospital inpatient care in Mongolia	Uranchimeg Ts et al	Article
Mongolian documents	2001	Medical education	Sumberzul N	Book
	2005	Social issues of Education in the Transition Period in Mongolia	Sarantuya O	Book
	2007	Evaluation of Medical Professionals' Licensing System in Mongolia	Ganbat B, Uranchimeg D	Article
	2009	Transitional period and legal reform in Mongolia	Amarsanaa J	Book
	2012	Quality evaluation of higher education in Mongolia	Tserendagva D et al	
	2012	Quality development of Medical education service: Experiences of Health Sciences University of Mongolia	Otgonbayar R et al	Article
	2016	Medical Education	Lhagvasuren Ts	Book
	2018	History of Mongolian 1911–2017	Batsaikhan O et al	Book

### Interview

We developed the interview questions based on the literature review, and conducted semi-structured interviews with local representatives. Question 4, 5, 6, and 7 were developed to check the controversy ideas from document review; question 2 and 3 were to validate the ideas and question 8, 9, 10, and 11 fill the information gap in the document review (Appendix 2). Three local researchers (NA; OB; and OR) purposively selected participants in accordance with the MLP framework (Table 3). The size of the sampling was inductively established and continued until data saturation occurred.

**Table 3:** Characteristics of Interviewees

Participant	ID	Gender	Current position		Graduation year
Decision-makers (MNUMS)	DM1	M	Executive committee member 1		1980–1990
	DM2	M	Director of academic center 1		1990–2000
	DM3	F	Executive committee member 2		2000–2010
	DM4	F	Director of academic center 2		2010–2020
Faculty members (MNUMS)	F1	F	Faculty of clinical department 1		1980–1990
	F2	F	Faculty of clinical department 2		1990–2000
	F3	F	Faculty of clinical department 3		2000–2010
	F4	M	Faculty of clinical department 4		2010–2020
Doctors	Do1	F	Doctor at a tertiary hospital		1980–1990
	Do2	F	Doctor at a tertiary hospital		1990–2000
	Do3	F	Doctor at a secondary hospital		2000–2010
	Do4	F	Doctor at a primary hospital		2010–2020

*MNUMS* Mongolian National University of Medical Sciences

We selected medical graduates from the Mongolian National University of Medical Sciences (MNUMS), which is the largest and only public medical school in Mongolia. MNUMS was established in 1942, and was the only medical school in Mongolia until the first private medical school was established in 1994 [17]. In accordance with the MLP framework, we included participants from diverse groups, consisting of decision-makers, faculty members, and doctors. Decision-makers provided relevant insights to the input level, as they are responsible for planning and management. Faculties members provided insights on the process level, as they deliver educational services, and doctors provided insights on the output level, as they are the products of the HPE system.

In accordance with the concept of time linkages in the MLP, we recruited participants from different graduation years. We divided them into four generations. First, the socialist HPE system existed until the 1990s. Second, the initial political and economic transition period continued through the 2000s, during which period the number of private medical schools dramatically increased. Third, the introduction of HPE reforms such as institutional accreditation, a licensing exam, and establishment of an integrated curriculum took place in the 2000s. Fourth, the development of the academic system has continued since 2010. National accreditation of the medical curriculum was introduced in the 2010s in order to improve the quality of private medical schools, and MNUMS was accredited by the Association for Medical Education in the Western Pacific Region.

Data collection and data analysis were conducted concurrently. We purposively selected the local representatives who have experiences in the field of HPE. We achieved data saturation at the 12th semi-structured interview, in other words, until no change to data were identified. According to the Romney, if the interview participants have a certain degree of expertise or knowledge on the research topic, data saturation can occur earlier on in interviews [18]. All the interviewers were female and medical doctors, who currently work in the field of health professions education. The main researcher was NA, who had 3 years of experience in the semi-structured interviewing. The participants were contacted primarily through the email, and none of them refused neither dropped out of the study. The researchers conducted the face to face and online interviews, which took from 50 to 90 min. The interview place was selected by the participants, and most of them were done in the workplace. Data were audio-recorded.

### Data analysis

We used qualitative content analysis and the constant comparative method to analyze the data. Two researchers in the field of HPE, including one local researcher (NA) and one expert (HBY), condensed the raw data. The researchers read through the data multiple times and underlined the content related to each level of MLP framework; they repeated the process until the new content could not been found. All transcribed-context units for each level were fully agreed upon by the researchers and placed into an Excel document. The third researcher (JSS) participated in quality assurance of the previous step and helped to develop explanations of the context units. Each researcher read the transcriptions multiple times and reached an agreement on the explanations of the context units.

### Ethics statement

The present study protocol was reviewed and approved by the Institutional Review Board of Seoul National University College of Medicine (approval No. 2002-118-1103). Informed consent was provided by all subjects when they were enrolled.

## Results

After the transition, Mongolia introduced local authority and privatization, reflecting similar directions to the reforms in other former socialist countries such as the Czech Republic and Poland [11]. These reforms affected the input level by impacting the governance authority and financing of the medical schools. On the process level, effects appeared in the learning environment and human resources. Effects on the output level appeared in the professional values and development of health professionals (Table 4). In the following sections, we discuss changes in these concepts by comparing the socialist and market eras.

**Table 4:** Effects of the transition

HPE system	Themes	Effects of transition
Input level	Governance authority	Academic authority without proper regulation
		Increasing number of unqualified private medical schools
	Financing	Lack of external financial support for medical schools
		Tuition fee-based revenue of medical schools
Process level	Learning environment	Absence of the core curriculum
		Varying quality of the learning environment
	Human resources	Poor environment for faculty development
		Poor clinical instructional supervision for specialized training
Output level	Professional development	Poor structure of licensing system
		Poor quality of continuing education
	Professional values	Lack of motivation due to financial pressure
		Decreasing public trust in health professionals

### Input level

#### Socialist era

The socialist system had centralized planning of human resources for health, based on a governmental assessment of the country’s situation. The Ministry of Health (MoH) set the admission quota for medical school and assigned workplaces for medical graduates based on the social needs of the population [14]. Moreover, the system fostered close cooperation between MNUMS and hospitals under the MoH, as hospitals provided large clinical bases to MNUMS [17]. The government fully funded MNUMS, so it faced no financial pressure. However, the expenditures on HPE were low due to the economic situation, and cooperation with non-socialist countries and availability of other financial sources were limited due to the political situation in Mongolia.

“Human resources for health planning in the socialist era was based on social needs” (F1)

“The medical school rigidly followed the rules to carry out planning without financial pressure” (DM1)

#### Governance authority in the Market era

After the transition, the government divided authority over human resources for health planning between the MoH and Ministry of Education, Culture, Sciences and Sport (MECSS). The MoH became responsible for health policies and hospitals [14], while the MECSS became responsible for educational policies and medical schools [19]. The absence of government regulation due to fragmented policies and weak management between ministries has resulted in quantitative imbalances of health professions [14]. For instance, hospitals received the authority to organize specialized training programs, which has resulted in an increasing number of specialists. Concurrently, the number of private medical schools dramatically increased without proper government regulation [20]. The licensure for private institutions based on the collection of documents rather than the social needs of the population [21]. In 1999, the government established the Department of Accreditation and Licensing Exams to ensure the quality of medical schools [22]. Tensions have existed regarding the quality of both the accreditation process and licensing exams [23]. Local stakeholders heavily criticized the increasing number of unqualified private medical schools, even though the accreditation system has existed for decades.

“Private medical schools are similar to business companies; conflicts of interest exist” (DM2).

“The accreditation process has not resulted in quality improvement so far” (F2).

#### Financing in the Market era

In 1997, the government decided to cover only the expenses of electricity and heating systems of higher education institutions [24]. Financial support from other countries has increased due to the new political structure, with the United States of America, South Korea, Japan, China, Great Britain, and Germany being the main providers [19]. However, most of these programs exclusively finance health improvements, rather than HPE as a part of the health system [11]. The absence of university hospitals limits the revenue of medical schools, and tuition fees are the only source of financial support of many medical schools. However, the amount of tuition fees continues to be assigned by the government; so that medical schools attempt to enroll more students in order to adapt in the market economy. Local stakeholders stated that the government should increase the authority of medical schools to regulate their finances and support them in efforts to find additional resources to improve their academic goals.

“Medical schools are completely dependent on tuition fees” (DM3).

“Government support, such as building university hospitals and supporting international projects, is needed for medical schools” (F3).

### Process level

#### Socialist era

Because of the close cooperation between MNUMS and hospitals in the socialist era, the learning environment for clinical practice was sufficient [17]. However, all aspects of the HPE system were dependent on political doctrine, to the point that half of the content of the medical curriculum was dedicated to the history of the Communist Party and the theory of communism [25]. Furthermore, the strong curative orientation of the health system and medical curriculum became non-responsive to social needs in the later years of socialism [26]. Medical graduates with higher scores were assigned to work as faculty members at MNUMS [27]. The government evaluated whether MNUMS fulfilled its annual plan, as well as individual whether individual faculty members completed their assigned tasks. The government provided performance-based incentives such as international training in different countries, vouchers for nursing camps, and opportunities for public housing.

“In the socialist era, cooperation between hospitals and MNUMS was strong” (DM4).

“Human resources were fully mobilized in accordance with planning” (Do1).

#### Learning environment in the Market era

After the transition, the quality of educational services varied across institutions [20]. The absence of a core curriculum resulted in varying quality of medical graduates from public and private medical schools. Moreover, the quality of specialized training programs varies across hospitals and medical schools; both programs have advantages and disadvantages. For instance, students trained at hospitals have more opportunities for clinical practice and are guaranteed employment at hospitals; however, the contents of these program do not correspond to global standards. In contrast, students at medical schools follow a standardized program, but, the environment is not supportive and opportunities for clinical practice are limited due to the absence of university hospitals. Factors such as enrollment number and educational services have not been unified across both types of programs [14].

“The development of a core curriculum through the cooperation of stakeholders is urgently needed” (F4).

“The involvement of hospitals in specialized training is increasing; it leads to conflicts between hospitals and medical schools” (DM1).

#### Human resources in the Market era

The rapid expansion of curricula and programs has led to increasing demands for faculty development [28]. International training and revolutions in information technology have provided opportunities; however, faculty members are struggling to keep pace with these challenges due to the insufficiently supportive environment [14]. For instance, clinical faculty members do not have opportunity to engage in clinical care due to the absence of university hospitals. Simultaneously, the supervisors of specialized training programs at hospitals are clinical physicians, most of whom do not have experience with educational strategies. Moreover, new aspects of the socio-economic environment have placed financial pressures on faculty members [3], who receive no financial compensation for research or extracurricular activities.

“Some clinical faculty prefer to work as clinicians due to the poor supportive environment of medical schools” (F1).

“Without proper assessments, it is difficult to pursue research as a primary goal” (F2).

### Output level

#### Socialist era

Because of the strong orientation towards curative services in the socialist era, MNUMS prepared graduates to be general doctors, pediatricians, dentists, and hygienists [27]. Medical graduates were highly experienced in clinical skills and could provide specialized healthcare services after graduation [17]. Health coverage was universal, even though services were not well resourced and equipped [27]. Furthermore, health professionals were motivated by factors such as guaranteed employment and public housing. The health professions were among the few high-class occupations at the time [25], and they retained considerable prestige in society.

“Final-year medical students used to work as clinicians in hospitals” (DM2).

“Patients and the public respected even medical students in the socialist era.” (Do2).

#### Professional development in the Market era

After the transition, the numbers of unqualified medical graduates increased, and health professions faced difficulties in maintaining professional development. All medical graduates should pass the licensing exam and obtain a 5-year license. In order to extend the license, health professionals must take the exam again or collect sufficient continuing education credits within 5 years [17]. However, Mongolia has still faced mismatches of competencies and insufficient capacity of health professionals [14]. Therefore, it is thought that licensing system has not been effectively implemented. First, the test-based structure of licensing exam conflict with the need to assess core competencies [22]. Second, collecting credits is more likely to be a system of control than to be a system aiming at improving the quality of health professionals.

“The test-based licensing exam assesses only the knowledge of health professionals” (F3).

“Collecting credits are not helpful for quality improvement” (Do3).

#### Professional values in the Market era

Another issue is that the fixed salary established by the government and the absence of incentives have undermined the motivation of health professionals. Concurrently, public trust in the healthcare system and health professionals is rapidly falling in Mongolia. Compared to the 1990s, the rising incomes of the population have resulted in increasing expectations for higher-quality healthcare services [17]. The lack of supplies and outdated equipment have impaired the diagnostic capacity of health professionals [14]. Local stakeholders mentioned that the health professions have not completely lost their perceived value due to the financial situation; therefore, a proper payment system and a supportive environment are needed.

“A performance-based payment system is crucial for motivation and professional development” (DM3).

“The government should prioritize the health sector and health professions in national policy” (Do4).

## Discussion

Many studies in social development research have focused on a single level, and the one sidedness of much social research has limited researchers’ ability to obtain deeper insights into the multiple levels of communities [15, 29]. We addressed the specific characteristics of multiple-levels of HPE system in the transition process; transition in the three level have direct and indirect influences on each other. Academic authority without proper regulation and increasing number of unqualified medical schools have resulted in the varying quality of learning environment and educational services. As a consequence, the competencies of health professionals have varied across institutions. Simultaneously poor structure of licensing system has not resulted in the quality improvement of health professionals; thus, public trust is decreasing. Concurrently, lack of financial support and poor supportive environment has led to the decreasing motivation of both faculties and health professionals. The implementation of reforms did not result in effective performance because of various unmet conditions [3]. As a consequence, Mongolia continues to face challenges in the healthcare and HPE systems.

Local authority was introduced without a supportive environment or proper regulation in Mongolia. The WHO has stated that decentralization is an ongoing process in many countries [30]; however, other researchers noted that central budgetary financing and hierarchical organizational structure continues to exist in the former socialist countries [31]. Local medical schools have the authority for service delivery, but not control over their finances [30]; the government sets tuition fees in Mongolia, and many local stakeholders of medical schools highlighted the difficulties caused by medical schools lacking authority over their finances. Moreover, the absence of the university hospitals poses tremendous challenges to medical schools, because it both limits their revenue and restricts the clinical learning environment. Thus, governmental support, such as building university hospitals and supporting international projects, is needed for the medical schools of Mongolia.

Simultaneously, rapid privatization was carried out without a competitive environment or a quality control system [11]. Thus, the number of unqualified medical schools is rapidly increasing in Mongolia. A study on South Asian countries showed that the majority of the private schools are for profit and similar to the “commercial” medical schools defined by Flexner [32]. The government of Mongolia established an accreditation system to assure the quality of medical schools [33]. However, regardless of whether an accreditation system exists, most countries have experienced tensions regarding the concept of accreditation. In order to attenuate such tensions, stakeholders within a country should clearly understand the nature of accreditation, including the educational intent of each standard in medical schools [34].

All actors, including government, health, and educational organizations, must jointly participate in the decision-making process to develop the HPE system [35]. However, there is no government policy in Mongolia to coordinate the separate activities of different organizations. The quality of educational services varies due to the different learning environments across institutions. The absence of a core curriculum has led to discrepancies in the competencies of health professionals [17]. Thus, local stakeholders recommended that private and public medical schools should collaborate to develop a core curriculum in Mongolia. Tensions regarding the curriculum are common at medical schools, and the curriculum from one school may not be directly applicable to another school. To implement innovation successfully, medical schools must consider the involvement of all actors, including students and faculty members, to encourage changes and to foster a supportive environment [34].

A study in Central and Eastern Asian countries showed that transition was followed by feelings of instability, lethargy regarding processes, and beliefs in social inequality [36]. In Mongolia, public trust in the healthcare system is falling. Socioeconomic developments and advances in medical technology have increased demand for various services [30]. However, the test-based licensing exam does not ensure the core competencies of health professionals; moreover, the licensing system has been oriented towards government control, rather than quality improvement of health professionals. Therefore, it is urgently needed to improve the licensing system for further quality improvement of health professionals. Some researchers recommended experimenting new strategies or policies based on the experiences of Chinese government, which was recognized by its rapid development and avoidance of the policy errors. The effectiveness of the new strategies could be defined by the policy experimentation on the small scale before the adoption at the national level. [37].

Furthermore, the-government-fixed salary and the absence of an incentive system lead to a lack of motivation for health professionals. Financial burdens contribute to low levels of motivation, and the type of the payment system has direct and indirect influences on the performance and motivation of health professionals [30]. Local stakeholders highlighted the need to introduce a performance-based payment system in Mongolia. A study in low- and middle-income countries showed that there is no standard blueprint for performance-based payments. The effectiveness of such systems may depend on the design of the payment system and specific aspects of the local situation [38]. Therefore, local stakeholders should consider starting conditions, the policy development process, design features, implementation, and the effects on health systems when introducing performance-based payments [39].

There are certain limitations of this study that should be considered. First, it is difficult to generalize the findings of a case study. However, a case study is a logical design for conducting in-depth research, and it is useful in the field of education to set standards by exploring past experiences and the development of new regulations. If the research employs sufficiently diverse approaches to data collection, such as interviews with diverse group of people and the analysis of documents from a broad range of sources, the conclusions are more likely to be applicable to other situations [40]. We hope that our framework could build a bridge between what has worked in Mongolia and how it could be applied to other transition economy countries. Second, the small number of participants likely resulted in a limitation of scope in terms of the issues addressed. To address this limitation proactively, we attempted to include participants from multiple groups, including decision-makers, faculty members, and doctors. The inclusion of participants from different groups helped us to find wide range perspectives on the local situation. We also recruited participants from different generations, who had collectively experienced the entirety of the socio-economic transition.

## Conclusion

Mongolia continues to face the consequences of the socio-economic transition. The lack of authority of medical schools over their finances, variation in the educational services offered by different organizations, and poor professional development of health professionals are the major adverse effects of the transition. First, the government should support medical schools through the additional funding opportunities, such as building university hospitals and participating in international projects. Second, collaboration among key stakeholders to develop a core curriculum is an urgent need. Quality assurance of the accreditation process and licensing system is also needed to improve medical schools and health professionals. Third, local decision-makers should consider how to maintain the value of health professions in a labor-intensive market. An effective way of doing so might be the reforming the payment system. This study contributes to shared learning in terms of improving strategies of HPE in transition economy countries. Future researchers and local decision-makers could consider these findings as they investigate and make decisions about the future directions of the HPE system.

## Figures and Tables

**Fig. 1:**
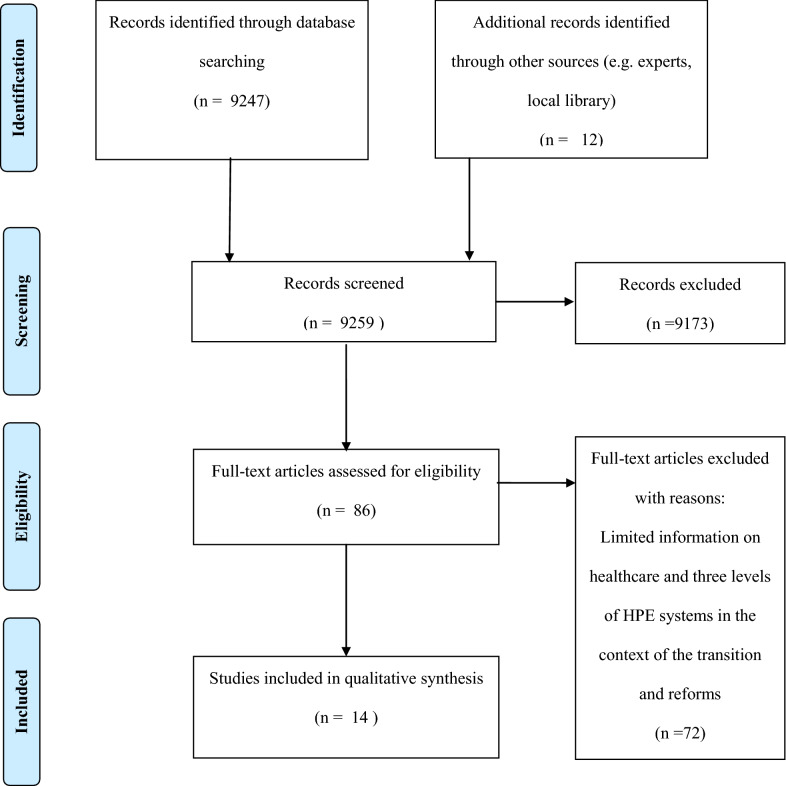
PRISMA flow diagram

## Data Availability

The dataset used and analyzed in the current study are available the corresponding author on reasonable request.

## Appendix 1

See Fig. 1

## Appendix 2. Interview questions

1. Please summarize your career experience thus far.
2. What are the current challenges in the health professions education (HPE) system in Mongolia?
3. What could be the root causes of the challenges faced by the HPE education system?
4. How does the reduction of government authority influence the HPE system?
5. How do government reforms such as the accreditation and licensing exam reflect the quality of educational services of HPE institutions?
6. How did the transition from the centrally planned economy to the market economy influence the HPE system?
7. What were the changes in the role of hospitals on the HPE system?
8. What were the behavioral changes of the public and patients in the market era compared to the socialist era?
9. How did the attitudes of health professionals change in the market era compared to the socialist era?
10. What could be learned from the socialist HPE system in Mongolia?
11. What type of changes are needed for further development of the HPE in the rapidly developing market environment?

## Abbreviations

HPE: Health Professions Education
MLP: Multi-level perspective
MNUMS: Mongolian National Medical University of Medical Sciences
MoH: Ministry of Health
MECSS: Ministry of Education, Culture, Sciences and Sports

## Declarations

### Authors’ information

The main researcher was NA, who had three years of experience in semi-structured interviewing while pursuing graduate studies. Two researchers (OB, OR) are the experts in the field of research and currently working as professors in Mongolia. Two researchers (HBY, JSS) are the professors and experts in medical education and research; both of them had five years of experiences in working with Mongolia. SHL is the expert in the medical education and research field.
